# Extending nanoindentation testing toward extreme strain rates and temperatures for probing materials evolution at the nanoscale

**DOI:** 10.1557/s43577-025-00918-7

**Published:** 2025-05-21

**Authors:** Benoit Merle, Gabrielle Tiphéne, Guillaume Kermouche

**Affiliations:** 1 Institute of Materials Engineering, Mechanical Behavior of Materials, University of Kassel, Kassel, Germany; 2 Institute of Mechanics, Materials and Civil Engineering (iMMC), UCLouvain, Louvain-la-Neuve, Belgium; 3 Mines Saint-Étienne, Laboratoire Georges Friedel, UMR5307 CNRS, Saint-Étienne, France

**Keywords:** Nanoindentation, Hardness, High strain rate, High temperature, Creep

## Abstract

**Abstract:**

For the past 30 years, nanoindentation has provided critical insights into the microstructure–strength relationship for a wide range of materials. However, it has traditionally been limited to quasistatic testing at room temperature, which has hindered a holistic understanding of microstructurally induced deformation mechanisms and their dynamic evolution as a function of the temperature and strain rate. Over the past decade, the operational scope of nanoindentation has expanded dramatically. Temperatures up to 1100°C and strain rates as high as 10^+4^ s^−1^ and as low as 10^−8^ s^−1^ have become accessible. In addition, advanced techniques allow tracking microstructural evolution and corresponding changes in mechanical behavior during deformation under extreme conditions. These advancements have transformed nanoindentation into a versatile tool for comprehensive materials characterization, enabling high-throughput investigations under multimodal conditions.

**Graphical abstract:**

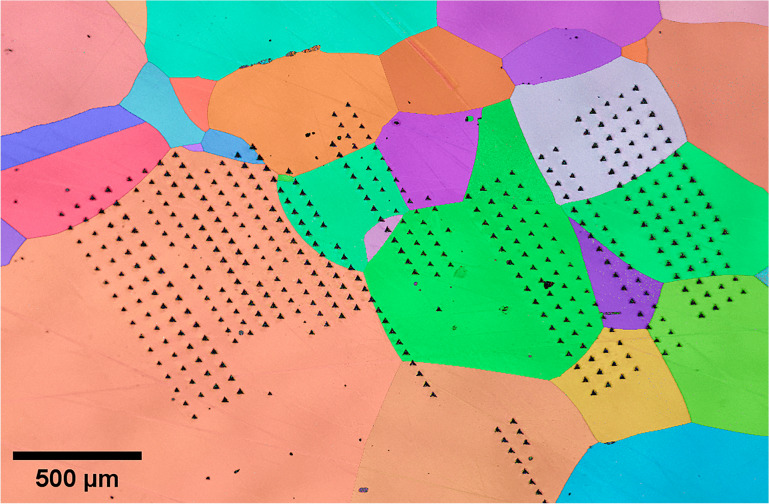

## Introduction

For the past 30 years, nanoindentation has been the method of choice for accessing the mechanical properties of materials on the micrometer-to-nanometer scale, with a strong focus on Young’s modulus and hardness.^1,2^ Nanoindentation has been successfully applied to all classes of materials and ultimately elevated to the ISO 14577 norm.^3^ Since then, the technique has experienced sustained further developments, which have considerably extended its scope of application. In this paper, we review the latest developments for investigating materials evolutions under extreme loading behaviors, which require spanning multiple orders of magnitude of strain rates and temperatures.

## Strain rate sensitivity probing

### Concepts

Creep is a deformation behavior associated with low strain rates that is typically found in materials continuously subjected to loads over long durations. Nanoindentation allows characterizing the associated deformation mechanisms by measuring the intrinsic creep flow parameters: strain rate sensitivity *m*, activation volume *V*, and activation energy *Q*. These materials parameters relate stress and strain rate within the framework of steady-state creep laws. A general form is given by the Garofalo equation:^4^

1 $$\upsigma =k {\text{sinh}}^{-1}\left({\left(\frac{\dot{\upepsilon }}{{\dot{\upepsilon }}_{0 }}\right)}^{m}\text{exp}\left(\frac{mQ}{RT}\right)\right),$$

where $\dot{\upepsilon }$ is the strain rate, $\upsigma$ is the steady-state stress, *T* is the absolute temperature, $m$ is the so-called strain rate sensitivity, ${\dot{\upepsilon }}_{0}$ is a constant homogeneous to a strain rate, $k$ is a constant homogeneous to a stress, $R$ is the gas constant, and $Q$ is the apparent activation energy. If the argument of the inverse hyperbolic sine is small, Equation 1 simplifies to the widely known Norton–Hoff Power Law: $\upsigma =k {\left(\frac{\dot{\upepsilon }}{{\dot{\upepsilon }}_{0 }}\right)}^{m}\text{exp}\left(\frac{mQ}{RT}\right)$. Strain rate sensitivity *m*, activation volume *V*, and apparent activation energy *Q* are easily computed from $m={\left(\frac{\partial \text{ln}\upsigma }{\partial \text{ln} \dot{\upepsilon }}\right)}_{T}$; $V=RT{\left(\frac{\partial \text{ln} \dot{\upepsilon }}{\partial\upsigma }\right)}_{T}$ and $Q={\frac{R}{m}\left(\frac{\partial \text{ln}\upsigma }{\partial \left(\frac{1}{T}\right)}\right)}_{\dot{\upepsilon }}.$

The calculation of creep parameters from nanoindentation data requires converting the hardness and indentation strain rate into a representative stress $\upsigma_{\text{r}}$ and a representative strain rate ε̇_r_. The existence of these representative parameters is a consequence of the principle of geometric similarity (PGS). It states that the stress and strain fields can be written as functions of dimensionless coordinates ${X}_{i }=\frac{{x}_{i }}{l(t)}$, where $l(t)$ is an indentation length scale (e.g. depth *h*, contact radius *a*), which depends upon time.^5,6^ Notably, only self-similar indenters, such as pyramidal tips, satisfy the PGS. The Tabor constraint factor *c* relates the representative stress to the hardness to: ${\upsigma }_{\text{r}}=H/c$. For a Berkovich tip, the constraint factor can be computed, for example, as $c\cong \left(\frac{0.243-0.783\frac{H}{E}}{0.087}\right)$ and converges toward $c \cong$ 2.8 for rigid plastic materials.^7^ The strain rate field is self-similar only if the indentation strain rate $\dot{\upepsilon }=\frac{\dot{h}}{h}$ is constant,^8^ as it is proportional to the actual representative strain rate:

2 $${\dot{\upepsilon }}_{r }\propto \frac{\dot{h}}{h}=\frac{1}{2}\frac{\dot{P}}{P}.$$

The astute reader may wonder how the representative strain rate can be nonzero while the representative strain under the indenter remains constant. A physical explanation lies in the increasing deformed volume with ongoing indentation,^9^ whereas a rigorous mathematical derivation is provided in Reference 10. From Equation 2, a constant strain rate (CSR) experiment requires an exponential increase of indentation depth *h* and/or load *P* with time: $h\left(t\right)={h}_{0}\text{exp}\left(\frac{\dot{h}}{h}t\right)$. It is worth mentioning that ${h}_{0}$ may not be equal to zero, that is, the constant strain rate condition can only be achieved after an initial loading segment, which is typically time-linear. With strain rate sensitive specimens, this initial loading introduces an artificial strain rate size effect at shallow depth.^11^

The CSR concept was first experimented by Lucas et al. on high-purity indium.^8,12^ The measured strain rate sensitivity and activation energy closely matched the uniaxial data.^13^ However, a shift along the strain rate axis was observed in the Norton plot ($\text{ln}\dot{\upepsilon }$ versus $\text{ln}\upsigma$), leading to the introduction of the so-called Bower correction factor.^14^ The Bower factor accounts for differences up to several orders of magnitude between ${\dot{\upepsilon }}_{\text{r}}$ and $\frac{\dot{h}}{h}$. The extension of Bower’s model by Ginder et al.^15^ further improved the accuracy. Kermouche et al.^7^ provided an explicit expression for indenting glassy polymers with a Berkovich tip:

3 $${\dot{\upepsilon }}_{\rm r }=0.16 \text{exp}\left(\frac{0.2}{m}\right)\frac{\dot{h}}{h}.$$

### Techniques

A proof of concept for abruptly changing the strain rate during one experiment was initially provided by Lucas and Oliver^8^ and later turned into the nanoindentation strain rate jump tests (SRJ) technique by Maier et al.^16^ SRJ now stands as one of the most popular methods to quantify the strain rate sensitivity and apparent activation volume of materials at the microscale. One clear advantage of SRJ compared to CSR is that the strain rate sensitivity can be computed from a single indent (i.e., the same specimen condition can be tested at different strain rates).

**Figure 1:**
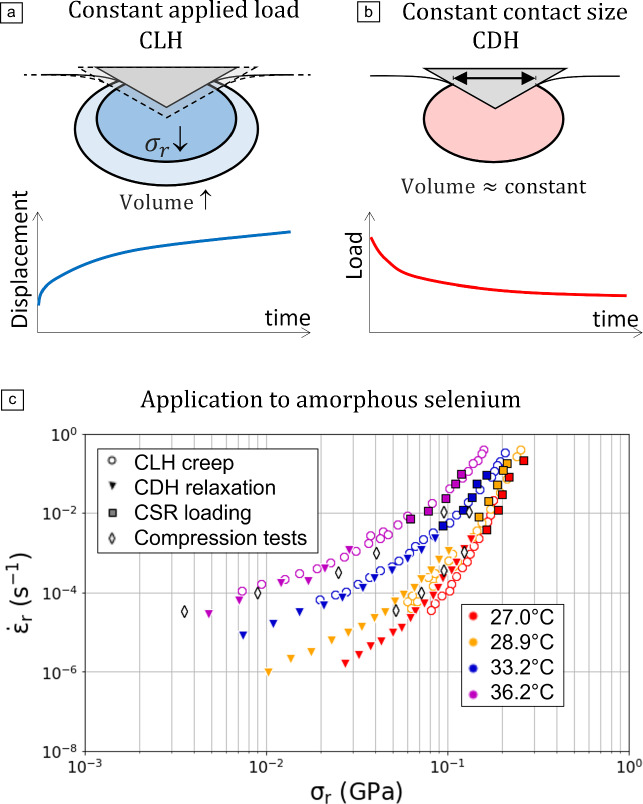
Schematics of (a) constant load hold (CLH) creep tests and (b) constant displacement hold (CDH) relaxation tests. Indentation creep tests are based on the measurement of the increase in indentation depth versus time during a constant applied indentation load segment. Indentation relaxation tests are based on the measurement of load decay during a constant contact size segment. (c) Indentation creep/relaxation behavior for amorphous selenium expressed in terms of representative stress and representative strain rate.^17^ CSR, constant strain rate.

Alternative techniques include indentation creep and indentation relaxation experiments. Indentation creep consists in applying a constant load hold (CLH) segment and measuring the increase in indentation depth and associated creep rate (**Figure** **1**a). Indentation relaxation experiments consist in holding the contact size constant (CDH: constant displacement hold) as depicted in Figure 1b, and measuring the corresponding load relaxation.

Although nanoindentation has been available for investigating the creep behavior of a wide range of materials,^14,15^ those that exhibit low creep rates can only be characterized by long-term creep experiments, which are impeded by the thermal drift issue. Maier et al.^18^ overcame the challenge by using continuous stiffness measurements (CSM), whereas the contact stiffness, *S*, is recorded continuously during the creep segment. Because CSM involves a fast oscillation, *S* is not affected by thermal drift, unlike the cumulative displacement.^19^ Both the hardness and the creep rate $\frac{\dot{h}}{h}=\frac{\dot{S}}{S}$ can thus be computed without detrimental influence from thermal drift. This method proved reliable for characterizing the creep properties of various materials, especially ultrafine grain metals. Its straightforward implementation made it one of the most popular techniques—together with SRJ—for measuring the strain rate sensitivity and activation volume.^20^

CLH nanoindentation differs from macroscopic uniaxial creep tests in a noteworthy aspect: The representative stress decreases continuously as the indenter sinks deeper into the surface during the indentation creep segment. This feature makes CLH more comparable to macroscopic relaxation testing than to conventional constant-stress creep testing. To address this issue, Minnert and Durst^21,22^ recently proposed an alternative methodology based on constant contact pressure holding (CCP). This condition is achieved by maintaining a constant $\frac{P}{{S}^{2}}$ ratio during the creep segment. CCP enables creep data measurements down to ca. 10^−6^ s^−1^. Aside from its implementation, which requires precise PID control loop parameters to maintain the ratio $\frac{P}{{S}^{2}}$ constant, the main limitation of this method is the significant increase of the indentation-affected volume during the creep segment. While this is also the case with CLH, CCP experiences a larger increase. Consequently, the material microstructure is never in a steady-state creep condition, as undeformed material keeps flowing inside the plastic zone.^22^

Such an issue can be avoided by using indentation relaxation experiments (constant displacement hold CDH). In this approach, the indentation-affected volume remains constant (Figure 1b) and the representative strain rate is computed as ${\dot{\upepsilon }}_{\rm r}=-\frac{1}{E}\frac{\text{d}{\upsigma }_{\rm r}}{\text{d}t}.$^23^ A key benefit of CDH over CLH creep and CSR nanoindentation is that the Norton plot ($\text{ln}\dot{\upepsilon }$ versus $\text{ln}\upsigma$) is obtained directly, eliminating the need for a shift factor. Conceptually, CDH could be conducted by maintaining the tip at a constant displacement, but this is only feasible in the absence of thermal drift. Baral et al.^23^ overcame this limitation by maintaining a constant contact stiffness—and therefore a constant contact area—instead of a fixed indentation depth, taking advantage of the robustness of CSM against thermal fluctuations. CDH enabled access to very low strain rates,^24^ down to 10^−8^ s^−1^, and was successfully applied to amorphous silica, PMMA and amorphous olivine.^25^ In amorphous selenium,^17^ CDH evidenced excellent agreement with indentation creep (CLH) and constant strain rate (CSR) nanoindentation (see Figure 1c).

### Comparison of CLH, CCP, and CDH

The techniques presented in this article have been shown to yield consistent creep parameters at room temperature. They can also be used at elevated temperatures to investigate the creep behavior of structural materials under application-relevant conditions. As discussed in the last section of this article, thermal drift effects become more pronounced at high temperatures, which is why high-temperature testing typically focuses on comparatively high strain rates.

Leaving aside high-temperature testing, a major difference between the techniques lies in the investigated strain rate range. Indentation relaxation experiments (CDH) allow investigating strain rates in the range from 10^−3^ to 10^−8^ s^−1^, whereas long-term indentation creep (CLH) provides information in the range from 10^−1^ to 10^−4^ s^−1^. Constant strain rate (CSR) and strain rate jump tests (SRJ) are better suited to characterize the creep behavior in a higher strain rate range up to 10^+4^ s^−1^. The challenges for performing CSR at high strain rates are discussed in the following section. Nanoindentation currently spans over 12 orders of magnitude in strain rates, as highlighted by Tiphéne et al.^24^ on CaF_2_. This strain rate versatility is a unique achievement for a single mechanical characterization technique.

## High strain rates

The mechanical behavior of materials, as well as the physical nature of the underlying deformation, changes drastically at high strain rates. A well-known example is the transition from dislocation-based deformation to viscous drag in metallic materials at ballistic speeds. As a result, standard datasheet mechanical properties are unsuitable for modeling structural behavior under shock loading or collision conditions.

Historically, nanoindentation has been limited to strain rates ≤ 0.1 s^−1^. This limitation has arisen from two primary reasons. First, the hardware components (controllers, sensors, and actuators) have traditionally been relatively sluggish, as high signal-to-noise ratio, robustness, and cost efficiency were often prioritized over speed. Additionally, at high strain rates, the measured signals are dominated by dynamic overload and further machine-related effects. Extracting meaningful sample data requires a well-behaved system along with precise modeling of its dynamic response. Second, the evaluation of nanoindentation data with the standard Oliver–Pharr method relies on the contact stiffness, which cannot be reliably measured under fast loading conditions.

### Fast nanoindentation hardware

The rapid advancement of gigahertz telecommunication signal processing in the 2010s has made high-speed electronic circuits widely available. The latest generation of nanoindenter controllers builds upon field-programmable gate array (FPGA) chips, which can reliably handle signals in the MHz range. Given the availability of these faster controllers, current efforts are increasingly focusing on improving the dynamics of sensors and actuators.

**Figure 2:**
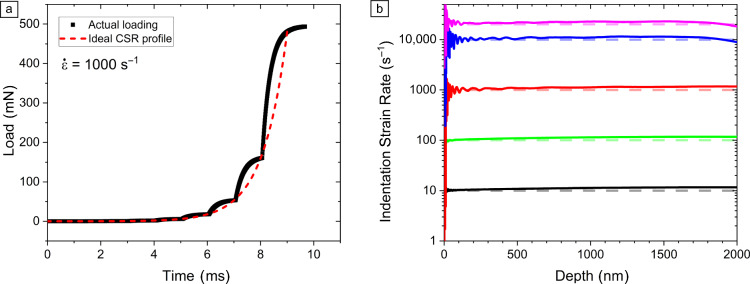
High strain rate nanoindentation testing: (a) effect of time constants and internal controller loop on force actuation during constant strain rate (CSR) nanoindentation with an instrument from the 2010s. (b) CSR nanoindentation with an FPGA-based controller on ultrafine grained aluminum up to 20,000 s^−1^. Notwithstanding the uncertainty of the measurements at shallow depths, the strain rates are effectively held constant.^26^

Assuming first-order linear time-invariant system behavior, the dynamic response of a sensor is characterized by its so-called time constant ${\uptau }_{\text{c}}$*,* which describes how quickly the sensor output matches the actual physical value to be measured (e.g., load, displacement). In detail, it takes a duration ${\uptau }_{\text{c}}$ for the output signal of the sensor to reach $\frac{e-1}{e}\approx 63.5\%$ of the physical value and $5{\uptau }_{\text{c}}$ to reach $99.3\%.$^26–28^ The same definition applies to actuators, where ${\uptau }_{\text{c}}$ describes how quickly the physical output approaches the set point (see **Figure** 2a). The time constant concept is closely related to the cutoff frequency (${f}_{\text{c}}$), a commonly used metric for electronic components:^29^

$${\uptau }_{\text{c}}=\frac{1}{2\pi \cdot {f}_{\text{c}}}.$$

Over the past decades, time constants of sensors and actuators have ranged between 1 and 100 ms.^30^ With the current generation of commercial nanoindenters, time constants as low as 20 µs have been achieved. On custom systems, further improvement by one order of magnitude has been achieved by switching to alternative sensors and actuators technologies. For displacement measurement, modern interferometers provide the best dynamic response^28,31^ (${\uptau }_{\text{c}}<1$ µs, compared to ${\uptau }_{\text{c}}\approx 20$ µs for the fastest capacitive gauge systems), but their accuracy is limited by the so-called cyclic error correction, causing ~3 nm noise.^32,33^ For force measurement, piezoelectric force cells offer the fastest response^31,34,35^ (${\uptau }_{\text{c}}<2$ µs, compared to ${\uptau }_{\text{c}}\approx 20$ µs for the fastest voice coil actuators), but suffer from charge leakage under static loads, leading to signal degradation within a few tenths of a second.^36^ This makes piezoelectric force sensors essentially unsuitable for indentation with strain rates below 10 s^−1^.

These technologies could see wider adoption in the coming decades. However, and as of now, the primary hardware limitation lies in the inertia and damping of the systems, which restrict the application of high loads at high displacement rates. At increasingly high strain rates, dynamic overload and machine effects dominate the measured response, overshadowing the sample contribution. While a correction—based on Newton’s Laws—is possible for both load-controlled and displacement-controlled systems,^35,37^ it requires a precise modeling of the dynamic response of the setup. Essentially, these corrections only hold up to a 10:1 ratio between machine and sample contributions.^28,38^

### Evaluation of high-speed data

The evaluation of experimental data using the Oliver–Pharr method and ISO 14577 requires knowledge of the contact stiffness.^1,33^ Recent findings indicate that the quasistatic unloading stiffness measured at peak force is susceptible to hysteresis, leading to misrepresentation of the actual contact condition at the investigated strain rate.^39^ For this reason, CSR testing is typically combined with the CSM technique, which provides a quasi-continuous evaluation of the contact stiffness during the loading segment, by superimposing a nanometer-scale oscillation.^40^ Although extremely useful, CSM introduces a critical limitation in terms of accessible strain rates. Indeed, the assumption that the CSM oscillations are purely elastic breaks down when load ramping becomes so fast that a significant part of a single oscillation experiences unprecedented loads and yields plastically.^41^ Under such circumstances, the measurements are affected by the so-called CSM plasticity error, which leads to an underestimation of the contact stiffness, as well as an overestimation of the hardness.^30,41,42^ With typical CSM parameters and metallic materials, the error on the determination of the hardness increases steeply at strain rates above 1 s^−1^, which can also be noticed from a spurious increase of the CSM phase angle.^30,41^ Custom systems have been operated at extreme CSM frequencies, as high as 1.5 kHz, which allows faster measurements, albeit only up to 10 s^−1^.^30^

At ultrahigh strain rates, the absence of direct stiffness measurement capabilities curtails the applicability of the Oliver–Pharr method. Specifically, the hardness cannot be computed because the contact depth ${h}_{\text{c}}$ is unknown. As a workaround, it was proposed to measure the so-called sink-in coefficient ${h}_{\text{c}}/h$ from slower indentations and assume that this ratio remains steady.^28,33^ This allows bypassing the contact stiffness and calculating the hardness from the measured displacement $h$. Unfortunately, experimental evidence shows that ${h}_{\text{c}}/h$ decreases when the hardness increases, which frequently occurs at high strain rates.^43^

The Merle–Higgins–Pharr (MHP) method provides an attractive alternative to standard evaluation techniques. Its core principle involves rearranging the equations of the Oliver–Pharr method and integrating prior knowledge of the invariable Young’s modulus *E* of the sample, which can be obtained from literature or from slower indentations, to eliminate the need for direct contact stiffness measurements.^26^ MHP has demonstrated strong consistency of the evaluated data with Oliver–Pharr, primarily because both methods rely on the same fundamental equations.

Next to these evaluation methods, different loading schemes have been implemented to perform measurements at high strain rates.

### Loading schemes for high strain rate nanoindentation

#### Nanoindentation impact testing

Nanoindentation impact testing traces its origins to the micro-impact setups developed to study the repetitive impacts experienced by hard coatings in machining operations.^44–46^ This technique has been applied to a wide range of nanoindenters and materials.^34,47–50^ A nanoindentation impact test typically begins with the indenter being retracted far from the surface and subsequently accelerated in the air before striking the surface of the sample at maximum velocity. In spring-retained indenters, the impact velocity can be maximized by backing to a given distance from the surface.^38^ This technique yields the highest achievable strain rates, on the order of 10^6^ s^−1^, albeit only for a brief duration and with a considerable uncertainty regarding the actual load applied to the sample, due to the initially dominant dynamic overload.^38^ As the indenter penetrates the surface, it decelerates and eventually rebounds, causing the strain rate to continuously decrease and even become negative. This raises concerns about possible hysteresis effects in strain rate-dependent materials, as the entire loading path influences the formation of the plastic zone and hence the final hardness values.^8,51^

Because contact stiffness cannot be measured during impact testing, early researchers replaced the Meyers hardness (Oliver–Pharr, ISO 14577) with an alternative energy-based definition, the so-called dynamic hardness. This definition correlates the kinetic energy dissipation during indentation to the impression volume before rebound. Unfortunately, the measured values deviate significantly from the Meyer hardness.^3,34^ This discrepancy likely arises from invalid assumptions—specifically, that hardness remains constant throughout the rebound and that no energy is dissipated by the indenter itself. A more promising approach involves applying the MHP method to impact test data to derive the Meyer hardness directly.

#### Constant strain rate nanoindentation (CSR)

An alternative to impact testing is CSR testing, an established technique that yet remains under active development. A significant benefit of CSR is that the measured hardness stems from a single strain rate, eliminating concerns over loading path hysteresis. Additionally, the effects of time constants and dynamic overloads are minimized, as the acceleration of the indenter is more gradual than with impact testing. Historically, the application of CSR to high strain rates was hampered by the impossibility to directly measure the contact stiffness. This limitation has now been overcome by the MHP evaluation method, so that the current bottleneck is purely hardware-related.^26^ On previous-generation nanoindentation systems, the accessible strain rate range was limited to ≤100 s^−1^ due to the kilohertz internal loop of the controller, which results in a discrete loading signal at high strain rates (Figure 2a). The newest FPGA-based controllers can sustain much higher strain rates (Figure 2b). The new limiting factor—expected at approximately 10,000 s^−1^—is primarily the mechanical behavior (damping and inertia) of the actuators and the associated dynamic overload.^33,35^

### Comparison to bulk techniques

Although the split Hopkinson pressure bar (SHPB) technique is widely used for characterizing homogeneous bulk samples, it is unsuitable for coatings and microstructurally complex materials. In contrast, high strain rate nanoindentation has recently been applied successfully to nanoscale metallic coatings^52,53^ and $\upgamma^ {\prime}$ precipitates from superalloys.^43^ A further benefit from nanoindentation is that the plastic zone is small compared to the sample dimensions, and the absolute velocity of the indenter remains low (typically <20 mm s^−1^) even for the highest strain rates. This makes the technique immune to issues arising from elastic wave propagation and adiabatic temperature increases, which often affect SHPB testing.^37,54^ This feature is particularly beneficial for combining high strain rate with high-temperature testing, for example, to investigate the behavior of the yield flow anomaly at high strain rates.^43^ Additionally, nanoindentation provides a wider range of accessible strain rates compared to traditional bulk testing methods.^55^ As a high-throughput technique, it also enables better statistical evaluations, leading to more reliable and reproducible conclusions.

## High temperature and high throughput

### Techniques and limitations

In metallurgy, heat treatments are widely used to tailor the mechanical properties of materials by introducing specific microstructural changes. Hardness testing and nanoindentation at room temperature (RT) are traditionally used to monitor these changes retrospectively after heat treatment.^56^ However, this approach is time-consuming and requires many samples to cover the full process temperature range. The process is streamlined by performing *in situ* testing at elevated temperatures in the range of interest.

The development of high-temperature nanoindentation started in the 1990s.^8^ Through iterative technical advancements, the maximum achievable temperature increased up to 1100°C.^57,58^ Building on these developments, *in situ* nanoindentation testing has been applied to a wide range of materials, delivering new insights into thermally induced transformations. Examples include the quantification of recrystallization kinetics in aluminum alloy at 300°C,^59^ oxidation of the sample,^60^ thermally induced phase transformations in NiTi shape-memory thin films,^61^ and the impact of precipitation aging on mechanical properties at high temperatures.^62^ Smith and Zheng^63^ showed that in silicon, the unloading pop-out associated with phase changes disappears at 200°C. Other studies explored the transition in deformation mechanisms related to the change in yield stress and the indentation size effect (ISE) across temperatures.^64–67^ Additionally, the stability of nanocrystalline materials, as well as their high-temperature properties, has been assessed.^20,68–71^ Some researchers have characterized the combined effect of ion implantation and temperature on the material behavior.^72,73^ Studies on metallic glasses,^74–78^ as well as polymers,^79–86^ have also been conducted at high temperatures. Finally, recent developments have allowed mapping the mechanical properties across a sample as a function of temperature.^87^ The same technique would allow investigating annealing-related changes in specific materials.

Performing indentation tests at high temperatures presents several challenges. First, the tip and sample temperatures must be precisely matched, and this should be ensured throughout the test.^88,89^ Any mismatch leads to thermal drift, resulting in inaccurate mechanical properties. At room temperature, thermal equilibrium is reached passively by allowing the system to stabilize in a closed environment for several minutes to hours. However, at elevated temperatures, passive stabilization is insufficient: Properly matching the heating systems on both sides is crucial.^90^ Failing to do so causes sudden heat transfer upon contact, leading to significant thermal drift. Heating up only the sample and transferring the heat through a long-term contact with the tip prior to indentation is not sufficient.^88,89^

A second challenge concerns the indentation tip. Nanoindentation analysis relies on accurate knowledge of tip properties and tip area calibration,^1,91^ meaning the tip geometry must remain stable throughout testing. Unfortunately, at high temperatures, the tip could dissolve into the sample or react with the testing environment,^92^ particularly during long-duration tests.^58^

Finally, precise control of the testing environment is essential. Not only does the environment affect the integrity of the tip, but it can also alter the surface chemistry of the sample.^24^ In order to ensure accurate characterization, oxidation kinetics may need to be quantified at specific temperatures, or oxidation may need to be entirely prevented through the use of vacuum.

### High-temperature scanning indentation (HTSI)

High-temperature scanning indentation (HTSI)^93,94^ was developed to overcome the thermal drift issue commonly encountered during long (minutes to hours) indentation tests at high temperatures. The key principle behind HTSI is that short indentation cycles minimize thermal drift effects: An individual cycle lasts for ca. 1 s, during which the temperature can be considered constant. This allows performing measurements not only during the annealing treatment at a given temperature, but also during heating and cooling of the system. With this, HTSI achieves a much higher temperature resolution than conventional high-temperature nanoindentation, which is typically performed at several discrete temperatures.

Each 1-s indentation cycle is designed to measure the hardness and Young’s modulus from the unloading segment, as well as the creep behavior from a short holding segment. By repeating this cycle continuously throughout the heating/cooling process, mechanical properties can be measured as a function of temperature without significant influence from thermal drift.

For materials exhibiting indentation size effects (ISEs), an improved load-controlled cycle has been proposed, which ensures a consistent indentation depth across the tested temperature range.^24^ The load set point is predicted from previous tests, assuming that hardness variations between successive indents are limited.

HTSI has been successfully applied to various materials: fused silica,^93,94^ pure aluminum,^93,94^ pure copper,^94^ and CaF_2_,^24,94^ for which it has demonstrated strain rate sensitivity measurements up to 800°C,^24^ with higher temperatures to follow up soon.^22^

The measured hardness, Young’s modulus, and creep properties were consistent with literature values, validating the technique.

Assuming that the tested material follows a Norton–Hoff Law, high-temperature HTSI data can be used to construct temperature-compensated strain rate versus modulus-compensated stress plots. If the material exhibits a single creep deformation mechanism over the studied temperature range, high-temperature testing at conventional strain rates is equivalent to low-temperature testing at extremely slow strain rates. This coupling between time and temperature allows indirect access to the creep behavior at extremely low strain rates ($\le\!\!{10}^{-9}$ s^−1^), which would take years to measure using conventional indentation creep tests at room temperature.^95^

A master creep curve was successfully obtained for CaF_2_, a material that is challenging to characterize at room temperature.^24^ The HTSI results aligned well with classical indentation creep and relaxation tests, as well as with literature.

Nevertheless, caution should be taken when interpreting HTSI creep data. As the creep segment is quite short, steady state may not always be reached. Additionally, at shallow indentation depth, the indentation size effect could introduce errors in strain rate sensitivity and activation volume.

### Applications in materials science

The new HTSI technique does not only speed up the characterization of the mechanical properties of a material as a function of the temperature, but also allows investigating microstructural and physical changes taking place dynamically during a heat treatment. Given that mechanical properties are inherently linked to microstructure, HTSI especially enables the characterization of microstructural evolution through real-time monitoring of mechanical changes.

**Figure 3:**
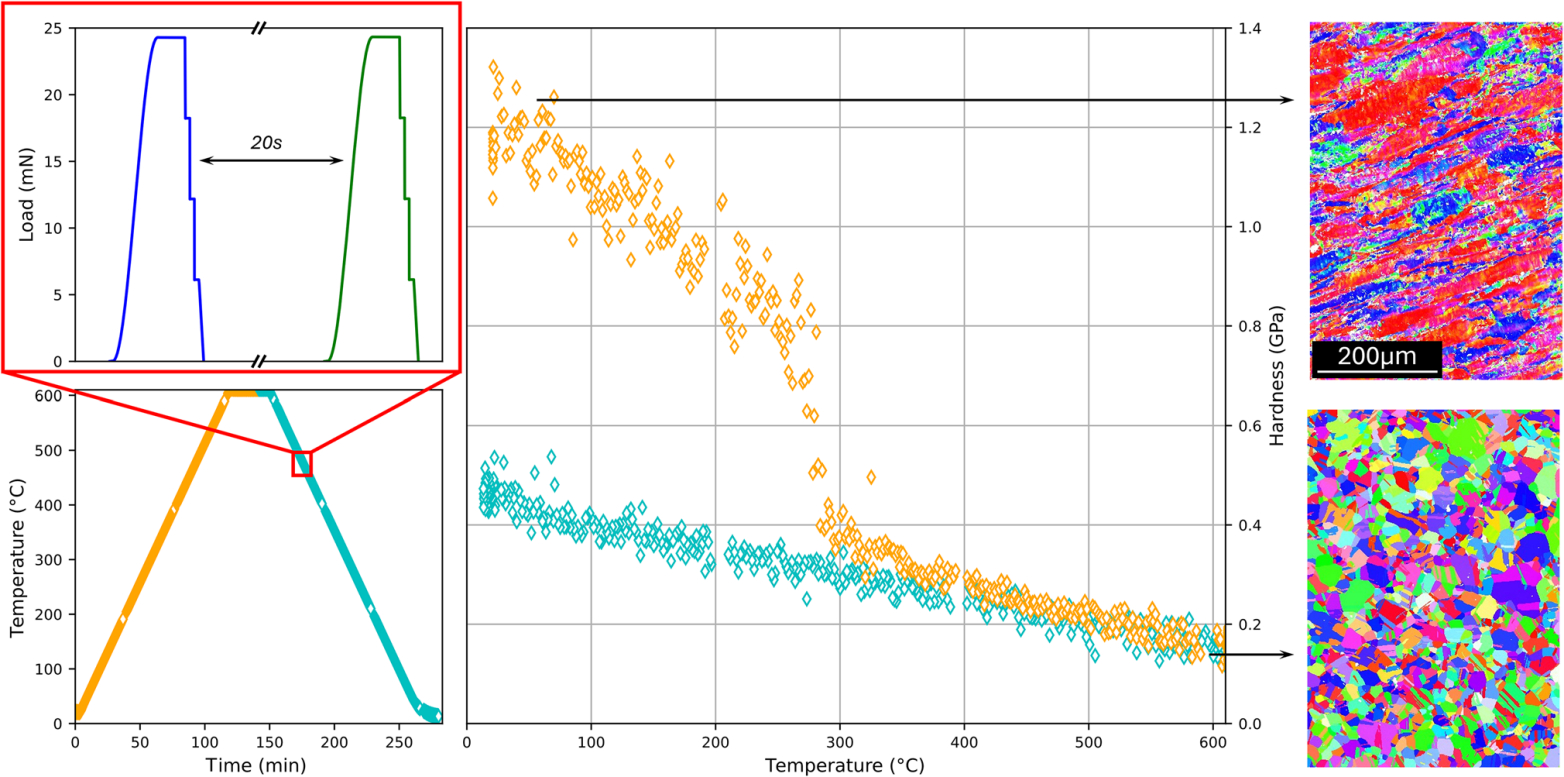
High-temperature scanning indentation applied to cold-rolled pure copper.^96^ The thermal cycle (bottom left) applied to the sample contains multiple 1-s indentation cycles (see excerpt, top left). The observed hardness drop at 250–300°C corresponds to recrystallization, as confirmed by electron backscatter diffraction (EBSD) analysis before and after testing.

For instance, in cold-rolled metals, HTSI allows for quantifying static recovery and static recrystallization, as demonstrated in studies on pure aluminum and pure copper.^94,96^ Because hardness and microstructure correlate, simple models can be used to describe hardness evolution with temperature, enabling microstructure predictions. An example can be found in **Figure** **3**: A copper sample is slowly heated from RT up to 600°C, while hardness is continuously measured. The observed drop in hardness at 250–300°C corresponds to recrystallization.

Another significant application of HTSI concerns metallic glasses. Such materials could be challenging to synthetize in bulk form, making nanoindentation an ideal technique to characterize them. Recently, HTSI was applied to ZrCu thin-film metallic glasses to investigate their behavior near the glass-transition temperature *T*_g_.^97^ From the changes in hardness and strain rate sensibility, the authors were able to determine the glass-transition temperature and observe the subsequent crystallization of the glass. Analysis of the pop-in events below *T*_g_ also provided insights into deformation behavior changes with temperature.

Finally, HTSI was also used to study the thermal stability of nanocrystalline metals.^98^ Changes in hardness and strain rate sensitivity revealed different thermal regimes. Depending on the maximum temperature reached, the microstructure either remained stable, or underwent minor or extensive grain coarsening.

Given its high versatility, HTSI has significant potential for materials science. Nanoindenter manufacturers are reportedly considering integrating it into commercial instruments, and its widespread adoption is expected to yield numerous new applications.^87^

## Conclusions

This article highlighted recent advancements in nanoindentation techniques for characterizing materials under extreme strain rates and temperatures. The latest developments have significantly broadened the scope of nanoindentation, enabling the measurement of hardness, Young’s modulus, and creep parameters—including strain rate sensitivity and apparent activation volume and energy—under previously inaccessible conditions.

Thanks to the recent advancements in high strain rate and creep testing, nanoindentation now spans 12 orders of magnitude of strain rates (between ${10}^{-8}$ and ${10}^{+4}$ s^−1^) at room temperature. This expansion has provided critical insights into the variability of deformation mechanisms. Ongoing developments seek to bridge the gap between experimental observations and computer-based molecular dynamic simulations (≥ ${10}^{7}$s^−1^).

Similarly, high-temperature developments have pushed the upper limit of nanoindentation to 1100°C, making it possible to study thermally activated processes and phase transformations. The recently developed high-temperature scanning indentation (HTSI) method streamlines testing and gives access to the coupling between microstructural, physical, and mechanical transformations.

Future directions include combining high strain rate and high-temperature capabilities to investigate complex deformation mechanisms under simultaneous extreme conditions.^43^ Low-temperature nanoindentation could also provide useful insights on materials for space and cryogenic applications. Additionally, integrating emerging techniques—such as electrical indentation^99–102^—will enable researchers to probe previously inaccessible phenomena and tailor materials properties for advanced applications.

In conclusion, the further evolution of nanoindentation will continue to unlock new possibilities for materials characterization, particularly for thin films and nanoscale phases. This will benefit both fundamental research and industrial applications, paving the way for the next generation of high-performance materials.
